# Physical Activity and Local Blue/Green Space Access During the COVID-19 Pandemic

**DOI:** 10.1177/08901171241244892

**Published:** 2024-04-05

**Authors:** Emily J. Nicklett, Bonita B. Sharma, Alexander Testa

**Affiliations:** 1 Department of Social Work, College for Health, Community and Policy, 12346 University of Texas at San Antonio, San Antonio, TX, USA; 2 School of Public Health, 12340 University of Texas Health Science Center at Houston, Houston, TX, USA

**Keywords:** physical activity, environmental support, blue space, green space, COVID-19, health promotion, social disparities

## Abstract

**Purpose:**

To examine whether local blue and green space access was associated with weekly physical activity frequency during the COVID-19 pandemic.

**Design:**

Cross-sectional.

**Setting:**

Population-based, nationally representative sample of U.S. adults (May and June 2021).

**Sample:**

Adults, ages 18-94 (N = 1,771).

**Measures:**

Self-reported data included the presence of blue spaces (e.g., lakes, outdoor swimming pools, riverside trails) and green spaces (e.g., parks, forests, or natural trails) in their neighborhoods, and days of physical activity per week (e.g., running, swimming, bicycling, lifting weights, playing sports, or doing yoga).

**Analysis:**

Multiple Poisson regression assessed relationships between blue and green spaces and physical activity, with coefficients transformed into incidence risk ratios (IRR).

**Results:**

Among participants, 67.2% reported living near a blue space and 86.1% reported living near a green space. Racial/ethnic and socioeconomic disparities in access to blue and green spaces were observed, with less access among non-Hispanic Black participants and those with lower income and educational attainment. Living near blue (IRR = 1.23, 95% CI = 1.10, 1.39) or green space (IRR = 1.25, 95% CI = 1.02, 1.54) was significantly associated with more frequent weekly physical activity.

**Conclusion:**

Proximity to blue or green spaces is associated with more frequent physical activity during the COVID-19 pandemic. Health promotion efforts should include equitable strategies to improve accessibility to blue and green spaces.

## Introduction

The COVID-19 pandemic has strained global health through increased morbidity, mortality, and social exclusion. Reduced physical activity levels during COVID-19 were associated with a significant population health impact,^1^ as the policies to contain the spread of the COVID-19 infectious virus included lockdown measures that restricted large indoor gatherings in the United States and other countries. One potential consequence of such measures was reduced opportunities for physical activity at indoor fitness centers and other wellness facilities. Indeed, research suggests that the measures to reduce the spread of the COVID-19 virus decreased human physical activity levels and increased daily sitting time by 28%.^2^ However, with the use of fitness centers and other wellness facilities limited during the COVID-19 pandemic, physical activity may have been more accessible to those who lived near outdoor areas that could be used for exercise, including green (i.e., parks, and trails) or blue (i.e., lakes, rivers, and beaches) spaces.

Access to green and blue spaces has emerged as a crucial factor in promoting physical and mental health.^3-5^ Limited studies have examined how greater access to green or blue spaces might promote physical activity, although existing studies conducted before the COVID-19 pandemic suggest that access to such spaces is beneficial for exercise and physical activity promotion.^6-9^ These environments offer benefits including opportunities for activities like walking, jogging, cycling, and other forms of outdoor physical activity,^10,11^ which have become essential alternatives to indoor fitness centers during the pandemic. Moreover, blue spaces have been associated with increased physical activity, as they offer opportunities for swimming, water sports, and walking along waterfronts.^12,13^ Blue and green spaces provide a refuge from chronic and acute psychological stressors^14^ and could, consequently, have motivated individuals to engage in physical activity for stress reduction, mental well-being, and physical health during the COVID-19 pandemic.

Despite the potential benefits of access to green or blue spaces on physical activity, there remains a lack of research into the possible relationship between access to green or blue spaces and patterns of physical activity during the COVID-19 pandemic in the United States. Even so, there are reasons to suggest that proximity to a green or blue space would be associated with more physical activity during the COVID-19 pandemic. Research has shown that the risk of virus transmission is lower in outdoor settings compared to indoor environments, primarily due to better ventilation and reduced close contact.^15^ This knowledge may encourage individuals to seek outdoor activities in green and blue spaces as a safer alternative to indoor settings, thereby increasing their engagement in physical activities.^16^ Additionally, access to these spaces supports the maintenance of social distancing measures while allowing individuals to connect with nature, improving their mental health and well-being during a time characterized by heightened stress and uncertainty.^3,5,17,18^

Using data from a nationally representative sample of American adults collected between May and June 2021, the present study examined whether living near a green or blue space during the COVID-19 pandemic is associated with more frequent physical activity.

## Methods

### Design

This was a cross-sectional survey of adults in the United States.

### Sample

This study used data from the 2021 Crime, Health, and Politics Survey (CHAPS), which is a national probability sample of 1771 community-dwelling adults 18 years and older living in the U.S. The primary purpose of the CHAPS is to document the social causes and consequences of health and well-being in the US during the COVID-19 pandemic. Respondents were sampled from the National Opinion Research Center’s (NORC’s) AmeriSpeak panel, which is representative of households from all 50 states and the District of Columbia.^19^ Sampled respondents were invited to complete the online survey in English between May 10, 2021, and June 1, 2021. The data collection process yielded a survey completion rate of 30.7% and a weighted cumulative response rate of 4.4% (which includes panel recruitment, panel retention rates, and survey completion rates). This cumulative response rate of 4-5% is within the typical range of high-quality general population surveys.^20^ The multistage probability sample resulted in a margin of error of ±3.23% and an average design effect of 1.92. While a margin of error of 3.00 is considered very good according to conventional practices, a smaller margin of error would indicate greater precision in estimates.^21^ The design effect of 1.92 is considered very good, indicating that the variance is only about twice as large as would be expected using a simple random sampling approach.^22^ Survey weights were included to reduce sampling error and nonresponse bias. This study was conducted according to the guidelines in the Declaration of Helsinki, and all procedures involving research study participants were approved by the institutional review board (IRB) at NORC and [removed] IRB. The current study used data from 1354 respondents with valid responses on the variables used.

### Measures

#### Physical Activity

The dependent variable is weekly self-reported *physical activity*, which is assessed in response to the question: “On how many days in a typical week do you take part in exercise activities like running, swimming, bicycling, lifting weights, playing sports, or doing yoga?”

#### Blue Space and Green Space

The independent variables are self-reported access to blue and green spaces in participants’ neighborhoods. *Blue space* is a variable based on a question asking, “Do you have access to any blue spaces in your neighborhood? By blue spaces we mean fountains, outdoor swimming pools, lakes, rivers, riverside trails, waterfronts, canals, seashores, or coastal areas?” (Yes or No). *Green space* is a variable based on a question asking, “Do you have access to any green spaces in your neighborhood? By green spaces we mean parks, gardens, mountains, natural trails, or forests with plants and trees?” (Yes or No).

#### Other Variables

Control variables measure various characteristics that may influence physical activity patterns, including respondents’ demographics, socioeconomic status, health, and COVID-19 history. Demographic measures included *age group* (18-29, 30-34, 45-59, or 65+), *sex* (0 = male, 1 = female), *race/ethnicity* (non-Hispanic white, non-Hispanic Black, Hispanic, other race/ethnicity), *born in United States* (0 = no, 1 = yes), and *marital status* ([a] married, [b] widowed/divorced/separated, [c] never married, or [d] cohabitating). Socioeconomic variables included *educational attainment* (less than high school, high school graduate/GED, some college, bachelor’s degree, or postgraduate study), *annual household income* (<$30,000, $30,000 to <$60,000, $60,000 to <$100,000, or ≥$100,000), and *household size.* We also controlled for whether a respondent lives in a *metropolitan area* (0 = no, 1 = yes) and whether a respondent *tested positive for COVID-19* (0 = no, 1 = yes). Finally, we controlled for a respondent’s *self-reported health* (poor or fair vs good, very good or excellent).

### Analysis

We first display the summary statistics of the analytic sample. Parametric *t*-tests were conducted to assess associations between sample characteristics and access to blue and green spaces, along with Cohen’s d values to determine effect sizes. Next, the bivariate relationship between physical activity days and living near a blue or green space is assessed using a two-tailed *t-*test. We then assessed the relationship between blue space and green space proximity and physical activity using multiple Poisson regression with coefficients transformed into incidence risk ratios (IRR). Finally, supplemental analyses are conducted to assess the interaction of green and blue space and how the results differ by respondents’ sex, age, race, and alternative classifications of the dependent variable. Statistical analyses included poststratification weights via iterative proportional fitting or ranking to general population parameters derived from the Current Population Survey. Poststratification weights were used to reduce sampling error and nonresponse bias. Standard errors were clustered on the state of residence.

## Results

Table 1 presents the summary statistics of the analytic sample. Overall, respondents reported exercising on average 2.56 days per week (standard deviation = 2.21; range = 0-7). 67.2% of respondents reported living near a blue space, and 86.1% reported living near a green space (t-score = 3.87, *P* < .001). Table 2 presents the sample summary statistics stratified by blue space and green space residence. In general, those who lived near blue spaces were older, less likely to be non-Hispanic Black, were married, had higher incomes, and smaller household sizes. Similarly, those who lived near green spaces were older, had higher educational attainment, were more likely to be married, had higher income levels, and smaller household sizes. The effect sizes (measured by Cohen’s d) generally suggest a small magnitude of differences between groups in bivariate analyses.

**Table 1.** Weighted Summary Statistics of Analytic Sample (N = 1354).

Variable	%/Mean	Standard Deviation	Minimum	Maximum
Dependent variable				
Physical activity days	2.56	2.21	0	7
Independent variables				
Blue space	67.2%		0	1
Green space	86.1%		0	1
Age				
18-29	18.1%			
30-44	26.1%			
45-59	23.2%			
60+	32.6%			
Female	51.7%		0	1
Race/Ethnicity				
Non-Hispanic White	66.4%		0	1
Non-Hispanic Black	12.9%		0	1
Hispanic	13.0%		0	1
Other Race/Ethnicity	7.8%		0	1
U.S. born	92.6%		0	1
Educational attainment				
Less than high school	7.8%		0	1
High school graduate/GED	31.0%		0	1
Some college	26.7%		0	1
College graduate	19.8%		0	1
Post-graduate	14.7%		0	1
Marital status				
Married	54.3%		0	1
Widowed/Divorced/Separated	18.6%		0	1
Never married	23.2%		0	1
Cohabitating	3.9%		0	1
Household income				
<$30,000	22.6%		0	1
$30,000 to Under $60,000	30.4%		0	1
$60,000 to Under $100,000	26.0%		0	1
>$100,000	21.0%		0	1
Household size	2.90	1.51	0	6
Metropolitan area	81.2%		0	1
COVID-19 positive	9.7%		0	1
Poor self-rated health	19.8%		0	1

**Table 2.** Weighted Summary Statistics of Analytic Sample by Blue Space and Green Space.

	Local Blue Space Access				Local Green Space Access			
	No (n = 454)	Yes (n = 900)			No (n = 178)	Yes (n = 1176)		
Variable	%/Mean	%/Mean	*P*-value	Cohen’s d	%/Mean	%/Mean	*P*-value	Cohen’s d
Dependent variable								
Physical activity days	2.11	2.78	<.001	.223	1.98	2.65	.014	.268
Age								
18-29	24.6%	14.9%	.011	.246	32.2%	15.8%	.008	.215
30-44	30.5%	23.9%	.061	.163	25.2%	26.2%	.813	.116
45-59	19.2%	25.2%	.59	.050	20.0%	23.7%	.382	.011
60+	25.7%	35.9%	.002	.282	22.6%	34.2%	.008	.250
Female	56.0%	49.7%	.113	.057	49.2%	52.2%	.607	.053
Race/Ethnicity								
Non-Hispanic White	62.2%	68.4%	.121	.165	60.0%	67.4%	.199	.207
Non-Hispanic Black	18.4%	10.1%	.012	.138	19.8%	11.7%	.127	.216
Hispanic	13.7%	12.6%	.686	.063	12.2%	13.1%	.802	.004
Other Race/Ethnicity	5.6%	8.9%	.123	.046	8.0%	7.8%	.927	.122
U.S. Born	93.7%	92.1%	.449	.037	94.3%	92.4%	.396	.020
Educational attainment								
Less than HS	7.8%	7.9%	.977	.0144	17.4%	6.3%	.069	.174
HS Graduate/GED	33.9%	29.5%	.280	.016	32.9%	30.7%	.685	.100
Some college	30.0%	25.1%	.122	.042	26.7%	26.7%	.998	.024
College graduate	16.3%	21.5%	.051	.021	14.3%	20.7%	.054	.048
Post-graduate	12.1%	16.0%	.110	.045	8.6%	15.7%	.012	.174
Marital Status								
Married	45.3%	58.6%	.001	.200	43.4%	56.0%	.025	.221
WDS	21.3%	17.4%	.212	.004	18.0%	18.8%	.823	.121
Never married	29.4%	20.2%	.017	.255	34.3%	21.4%	.033	.142
Cohabitating	4.0%	3.8%	.881	.022	4.3%	3.8%	.797	.023
Household income								
<$30,000	28.9%	19.6%	.013	.203	35.9%	20.5%	.009	.241
$30,000 to < $60,000	35.5%	27.8%	.041	.067	32.0%	30.1%	.715	.053
$60,000 to < $100,000	22.4%	27.7%	.096	.042	22.1%	26.6%	.295	.068
>$100,000	13.1%	24.9%	<.001	.229	10.0%	22.8%	<.001	.223
Household size	3.09	2.81	.037	.172	3.36	2.83	.016	.218
Metropolitan area	83.3%	80.1%	.267	.075	82.1%	81.0%	.776	.006
COVID-19 positive	12.4%	8.5%	.166	.107	15.8%	8.8%	.180	.168
Poor SRH	24.2%	17.6%	.060	.117	25.5%	18.9%	.243	.209

Note. HS = high school; WDS = widowed, divorced, or separated; SRH = self-rated health.

**Figure 1.**
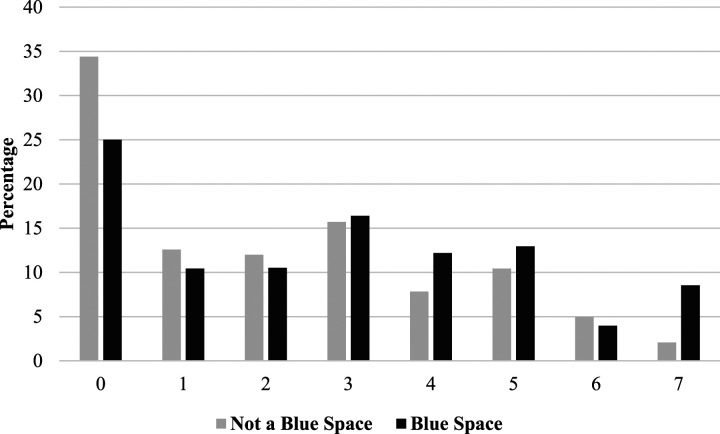
Physical activity days by blue space access.

**Figure 2.**
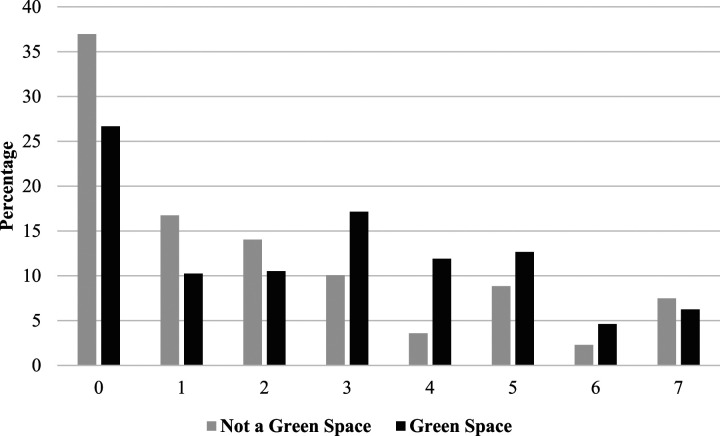
Physical activity days by green space access.

Figure 1 shows the distribution of exercise per day of the week among respondents who live near a blue space and those who do not. The distribution shows a higher prevalence of persons who exercise zero days among those who do not live in blue spaces and a higher prevalence of more days, especially 4, 5, or 7 days among those living near blue spaces. Those who live near blue spaces report physical exercise 2.78 days per week compared to 2.11 days per week among individuals who do not live near a blue space (t-score = 3.87, *P* < .001). The Cohen’s d effect size of .223 indicates a small, but statistically significant difference in physical activity between those residing near blue spaces compared to those who do not reside near blue spaces. Figure 2 details a similar pattern of less frequent exercise—particularly 0-2 days a week—being more prevalent among respondents who do not live near green spaces, but more frequent exercises—particularly 3-7 days a week—being more frequent among residents who live near green spaces. Respondents who lived near a green space reported an average of 2.70 physical activity days compared to 2.13 physical activity days for those who did not live near a green space (*t-*score = 3.204, *P* = .001). The Cohen’s d effect size of .268 represents a small, although statistically significant difference in physical activity when comparing respondents who reside near green spaces with respondents who do not reside near green spaces.

Adjusting for control variables, the results in Table 3 show that living near a blue space is associated with approximately a 23% higher rate of physical exercise per week (IRR = 1.23, 95% CI = 1.10, 1.39), and living near a green space is an associated with approximately a 25% higher rate of exercise per week (IRR = 1.25, 95% CI = 1.02, 1.54).

**Table 3.** Multiple Poisson Regression of Physical Activity Days on Blue Space/Green Space (N = 1354).

Variable	IRR	95% CI
Blue space	1.23***	(1.10, 1.39)
Green space	1.25*	(1.02, 1.54)

Note. Control variables include age, sex, race/ethnicity, born in the United States, educational attainment, marital status, annual income, household size, metropolitan area, COVID-19 positive, and poor self-rated health. Standard errors clustered on state of residence. Each row represents a separate Poisson regression model.

***p < .001, **p < .01, *p < .05.

### Supplemental Analyses

The supplemental analyses showed that there is no significant interaction between proximity to blue space and green space for physical exercise (Appendix A), by sex (Appendix B), or age (Appendix C). The results in Appendix D and E show that while living near a blue space is associated with more frequent exercise for all race and ethnic groups, this effect size is significantly strongest for Hispanic respondents, who have a predicted number of 1.46 physical activity days for those not in a blue space, compared to 2.71 days for those in a blue space. There is no significant difference for green spaces. Finally, Appendix F recategorizes the dependent variable into the following categories: 0 days, 1-2 days, 3-4 days, and 5 or more physical activity days. The findings from multinomial logistic regression models show that blue space proximity is significantly associated with physical exercise 3-4 days (RRR = 1.61, 95% CI = 1.03, 2.52) and 5 or more days per week (RRR = 1.80, 95% CI = 1.17, 2.78) relative to zero days. Additionally, the results show that proximity to a green space is associated with physical exercise 3-4 days per week (RRR = 2.62, 95% CI = 1.47, 4.71) relative to 0 days.

## Discussion

The findings from the current study using a nationally representative sample of adults from during the COVID-19 pandemic in May and June 2021 found that living near a blue or green space was significantly associated with more physical activity days. These results are consistent with prior literature detailing an association between blue and green spaces and healthier lifestyle behaviors, including more exercise, from childhood^6,9^ to older adulthood.^7,23^ The majority of these studies, however, have focused on specific age groups and were conducted outside of the pandemic or U.S. contexts. Among children specifically, prior studies have identified positive relationships between blue and green space access and physical activity. For example, an Australian study^6^ of 1216 children ages 8-13 years (conducted April-June 2015) found that girls living within 5 kilometers of the coast had higher mean moderate-to-vigorous physical activity minutes (measured through accelerometry) compared to girls living in towns ≥50 kilometers from the coast, while boys living within 5 kilometers of the coast were more likely to meet self-reported physical activity guidelines (≥60 minutes per day for ≥5 days) compared with boys living ≥50 kilometers from the coast. A study conducted in Germany in 2005 among children ages 3-19 years^9^ examined associations of the proportion of green spaces in the immediate urban living environment with outdoor activity, finding that higher proportions of green space were associated with greater outdoor activity in winter among older children (age 10-19).

Among older adults specifically, prior studies have identified associations between blue and green space access and physical activity participation. For example, a longitudinal cohort study^23^ of older adults in Norfolk, UK from 1993-2009 found when comparing people living in objectively measured “greenest” vs “least green” quartiles that participants showed a difference in overall physical activity of 4.21 MET hours/week, adjusting for baseline physical activity and sociodemographic covariates. Additionally, a qualitative study^7^ of adults ages 65-86 years in metro Vancouver, Canada in 2012-2013 found that nature plays an influential role in physical well-being, including motivating physical activity for recreational and purposeful exercise. The present study builds upon this prior literature on physical activity and blue/green space access as (1) among the first studies on this topic conducted during the COVID-19 pandemic; (2) to our knowledge, the first study on this topic using data from a national sample of U.S. adults; (3) to our knowledge, the first study to examine the roles of race/ethnicity and other key sociodemographic characteristics as potential moderators.

Local access to green and blue spaces allows populations to engage in free or low-cost physical activity while reducing transportation-related barriers. The benefit of green and blue spaces can be observed across neighborhoods and communities, with parks, lakes, and other public natural areas providing settings for recreational activities that also contribute to social support and community cohesion.^7,24,25^ As social support and community cohesion also independently contribute to physical activity participation,^26,27^ blue and green spaces provide communities with more than just opportunities to exercise: they provide a backdrop and setting for developing and sustaining health-promoting activities and relationships.

The spread of COVID-19 reduced opportunities for people to safely engage in traditional forms of indoor physical activity.^28^ For those who had access to them, outdoor spaces provided spaces for individuals to be physically active while avoiding the risk of spreading COVID-19.^29^ As climate crises and other human disasters continue to threaten population health, the cooling effects of blue spaces (lakes and springs) and shaded green areas should be increasingly prioritized in local and national disaster planning and prevention strategies.

As this study found that access to blue and green spaces was associated with more days of physical activity consistently across gender, age group, educational status, income group, and other sociodemographic characteristics, the benefit appears to be universal and could, therefore, be used to guide approaches to improve community and population health. However, it is worth emphasizing that not everybody has equal access to green and blue spaces. In the present study, participants with lower reported income and education levels were significantly less likely to have access to blue and green spaces. These findings are consistent with an ample body of literature finding socioeconomic disparities in blue and green space access.^30-34^ Additionally, participants who identified as Hispanic or as non-Hispanic Black were less likely to have access to blue and green spaces than participants who identified as non-Hispanic White. The racial and ethnic disparities in blue and green space access point to the ongoing impact of policies embedded in structural racism, such as redlining and other forms of racial residential segregation, as mechanisms continuing to underlie health inequalities in the United States.^30,35–41^ Public health initiatives to promote physical activity and other health-promoting behaviors must consider equitable strategies to improve access to blue and green spaces for population groups experiencing health disparities.

Despite the valuable insights provided by this study, several limitations warrant exploration in future research. First, the survey data collection took place from May 10, 2021, to June 1, 2021, offering a snapshot of behavior during the COVID-19 pandemic but covering only a limited period. Consequently, evaluating the relationship between blue space/green space and physical exercise during other pandemic phases is challenging. While this study sheds light on the association between blue space/green space and physical exercise patterns within this timeframe, future research should investigate how blue space/green space status relates to physical activity during other periods. Second, the measurement of physical activity participation, blue space access, and green space access relied on single-item, self-reported measures, which might be subject to self-report bias. We cannot capture details on the intensity or duration of physical activity participation or what type of blue (i.e., lake, swimming pool) or green (i.e., park, hiking trail) space a respondent resides near. In addition, we cannot discern how geographically far a respondent lives from the space or how often they utilize the space. Likewise, while there is a relationship between proximity to a blue/green space, we cannot discern whether the respondent specifically utilizes that space for physical exercise. A valuable avenue for future research involves gaining deeper insights into the underlying factors contributing to how blue space/green space contributes to physical activity patterns, including during the COVID-19 pandemic. Future studies could consider incorporating objective measures of physical activity and blue/green space access for more accurate results. Further, qualitative research conducted with individuals who do not utilize blue space/green space for exercise may provide valuable perspectives. Finally, the CHAPS is a cross-sectional survey, limiting the ability to establish causal relationships. Future research that uses longitudinal data that can detect changes in proximity to blue/green spaces and physical activity over time would be especially valuable. Future research could benefit from a longitudinal approach to better understand changes in physical activity patterns over time and in different phases of the pandemic.

In conclusion, individuals who indicated living near a blue or green space during the COVID-19 pandemic reported more frequent physical exercise concerns than those who did not live near such areas. These findings add to the results from extant literature highlighting the importance of the proximity of blue and green spaces for physical activity and build on this literature by showing the importance of these spaces during the COVID-19 pandemic when the ability to physically exercise through other means (i.e., gym memberships) were limited. These findings highlight the importance of investing in outdoor spaces that promote physical activities to improve the health and well-being of individuals in communities and search for alternative ways to promote physical activity in communities without access to such spaces.

**So What? Implications for Health Promotion Practitioners and Researchers**

**What is Already Known on This Topic?**

Access to blue and green spaces has been identified as a crucial factor in human health and well-being. However, no prior studies have characterized the association between blue and green space access and physical activity participation among U.S. adults during the COVID-19 pandemic.

**What Does This Article Add?**

This study found that access to blue and green spaces was associated with more frequent weekly physical activity. Moreover, racial/ethnic and socioeconomic disparities were observed in access to blue and green spaces.

**What Are the Implications for Health Promotion Practice or Research?**

Our findings highlight the need for community strategies and national policies to improve access to blue and green spaces, particularly in communities experiencing health disparities. As climate crises and other human disasters continue to threaten population health, the cooling effects of blue spaces and shaded green areas should be increasingly prioritized in local and national disaster planning and prevention strategies.

## Figures and Tables

**Appendix E.**
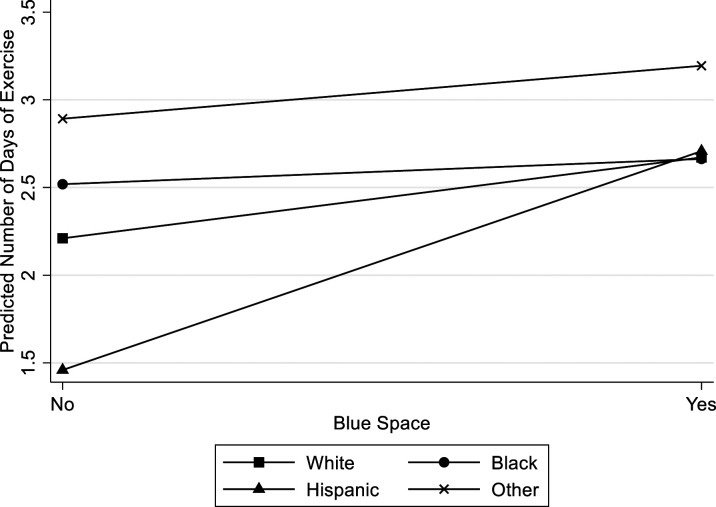
Multiple Poisson Regression of Physical Activity Days on Blue Space/Green Space * Race/Ethnicity (N = 1,354).

## Appendix

**Appendix A:** Multiple Poisson Regression of Physical Activity Days on Blue Space * Green Space (N = 1354).

Variable	IRR	95% CI
Blue space	1.22	(.92, 1.62)
Green space	1.30	(.78, 2.17)
Blue space * Green space	.90	(.53, 1.55)

Note. Control variables include: age, sex, race/ethnicity, born in the United States, educational attainment, marital status, annual income, household size, metropolitan area, COVID-19 positive, and poor self-rated health. Standard errors clustered on state of residence. Each row represents a separate Poisson regression model.

****p* < .001, ***p* < .01, **p* < .05.

**Appendix B:** Multiple Poisson Regression of Physical Activity Days on Blue Space/Green Space * Sex (N = 1354).

Variable	IRR	95% CI
Blue space	1.24	(.96, 1.58)
Female	1.04	(.83, 1.32)
Blue space * Female	1.00	(.73, 1.36)
Green space	1.30	(.95, 1.77)
Female	1.11	(.73, 1.68)
Green space * Female	.93	(.62, 1.39)

Note. Control variables include age, race/ethnicity, born in the United States, educational attainment, marital status, annual income, household size, metropolitan area, COVID-19 positive, and poor self-rated health. Standard errors clustered on state of residence.

****p* <.001, ***p* <.01, **p* <.05.

**Appendix C:** Multiple Poisson Regression of Physical Activity Days on Blue Space/Green Space * Age (N = 1354).

Variable	IRR	95% CI
Blue space	1.43*	(1.04 - 1.96)
Age: 30-44	1.05	(.77 - 1.43)
Age: 45-59	.87	(.56 - 1.33)
Age: 60+	1.21	(.83 - 1.76)
Blue space * Age: 30-44	.83	(.59 - 1.19)
Blue space * Age: 45-59	1.04	(.64 - 1.67)
Blue space * Age: 60+	.72	(.49 - 1.06)
Green space	1.02	(.68 - 1.55)
Age: 30-44	.87	(.52 - 1.48)
Age: 45-59	.47*	(.23 - .95)
Age: 60+	.83	(.50 - 1.40)
Green space * Age: 30-44	1.10	(.68 - 1.78)
Green space * Age: 45-59	2.16	(.97 - 4.82)
Green space * Age: 60+	1.22	(.71 - 2.09)

Note. Control variables include sex, race/ethnicity, born in the United States, educational attainment, marital status, annual income, household size, metropolitan area, COVID-19 positive, and poor self-rated health. Standard errors clustered on state of residence.

****p* < .001, ***p* < .01, **p* < .05.

**Appendix D:** Multiple Poisson Regression of Physical Activity Days on Blue Space/Green Space * Race/Ethnicity (N = 1354).

Variable	IRR	95% CI
Blue space	1.21**	(1.06 - 1.38)
Black	1.14	(.87 - 1.49)
Hispanic	.66	(.42 - 1.04)
Race/ethnicity	1.31	(.89 - 1.93)
Blue space * Black	.87	(.62 - 1.24)
Blue space * Hispanic	1.53*	(1.02 - 2.31)
Blue space * Other Race	.91	(.60 - 1.39)
Green space	1.16	(.95 - 1.43)
Black	.72	(.34 - 1.54)
Hispanic	.72	(.44 - 1.19)
Other race/ethnicity	1.41	(.81 - 2.46)
Green space * Black	1.55	(.69 - 3.50)
Green space * Hispanic	1.29	(.84 - 1.98)
Green space * Other Race	.86	(.48 - 1.55)

Note. Control variables include age, sex, born in the United States, educational attainment, marital status, annual income, household size, metropolitan area, COVID-19 positive, and poor self-rated health. Standard errors clustered on state of residence.

****p* < .001, ***p* < .01, **p* < .05.

**Appendix F:** Multiple Multinomial Logistic Regression of Physical Activity Days on Blue Space/Green Space (N = 1354).

	1-2 Days vs 0 Days		3-4 Days vs 0 Days		5+ Days vs 0 Days	
Variable	RRR	95% CI	RRR	95% CI	RRR	95% CI
Blue space	1.13	(.73 - 1.77)	1.61*	(1.03 - 2.52)	1.80**	(1.17 - 2.78)
Green space	.92	(.52 - 1.64)	2.63**	(1.47 - 4.71)	1.62	(.89 - 2.95)

Note. Control variables include age, sex, race/ethnicity, born in the United States, educational attainment, marital status, annual income, household size, metropolitan area, COVID-19 positive, and poor self-rated health. Standard errors clustered on state of residence. Each row represents a separate Poisson regression model.

****p* < .001, ***p* < .01, **p* < .05.
